# A Softsensor for Wind Measurements in Karst Caves

**DOI:** 10.3390/s26010022

**Published:** 2025-12-19

**Authors:** Juš Kocijan, Matija Perne, Franci Gabrovšek, Primož Mlakar, Boštjan Grašič, Marija Zlata Božnar

**Affiliations:** 1 Jožef Stefan Institute, Jamova cesta 39, 1000 Ljubljana, Slovenia; matija.perne@ijs.si; 2 Centre for Information Technologies and Applied Mathematics, University of Nova Gorica, 5000 Nova Gorica, Slovenia; 3 Faculty of Mathematics and Physics, University of Ljubljana, 1000 Ljubljana, Slovenia; franci.gabrovsek@zrc-sazu.si; 4 Karst Research Institute, Research Centre of the Slovenian Academy of Sciences and Arts ZRC-SAZU, 6230 Postojna, Slovenia; 5 MEIS d.o.o., Mali Vrh pri Šmarju, 1293 Šmarje–Sap, Slovenia; primoz.mlakar@meis.si (P.M.); bostjan.grasic@meis.si (B.G.); marija.zlata.boznar@meis.si (M.Z.B.)

**Keywords:** soft sensor, karst, cave, meteorology

## Abstract

A data-driven soft sensor of wind in a cave passage is developed as an alternative to physical anemometers for measuring wind velocity. It is intended to either fill data gaps during periods without physical measurements or to serve as a substitute for the physical sensor. It is implemented as a Gaussian process model, trained on one year of half-hourly measurements. Statistical measures and visual inspection of the test data indicate that both selected model structures perform well. Therefore, soft sensors represent a viable tool in underground meteorology. They may replace physical sensors that are fragile, power-intensive, or expensive. Alternatively, they can fill data gaps when a physical sensor is unavailable.

## 1. Introduction

The use of soft sensors offers excellent potential for studying karst underground air. We present an example of a successfully developed soft sensor of wind along a cave passage.

Karst is a type of topography formed on soluble bedrock and characterised by distinctive landforms such as caves. As a result of dissolution, karst contains extensive air-filled underground voids of large volumes and dimensions, which can extend to considerable depths below the surface. Several reasons exist to study the underground air within these voids. One application is the use of speleothems, cave mineral deposits, as paleoclimate proxies: the speleothem’s $\delta^{13}$C value reflects the $\delta^{13}$C value and dissolved inorganic carbon (DIC) concentration in drip water [1,2,3]. However, it also depends on the CO_2_ partial pressure $p{\mathrm{CO}}_{2}$ and isotopic composition in the cave atmosphere [1,2,4]. Thus, understanding the composition of cave air is necessary for climatic interpretation of the $\delta^{13}$C signal [2]. Interpretation of speleothem $\delta^{18}$O signals is analogous: they are influenced both by regional climate and by local cave conditions [5,6]. Karst underground air is also a significant source of radon in buildings [7,8] and responds dynamically to climate changes [9,10]. It provides insight into inaccessible parts of the karst subsurface [2,11]. Moreover, karst aquifers are an important water source, supplying an estimated 9 % [12] to 25 % [13] of the global population. Air conditions constrain the sustainable use of caves in tourism [14,15,16,17,18]. In carbonate karst, the dissolution reaction consumes CO_2_ [19,20] and is being investigated as a potential carbon sink [21,22]. In aquifer regions dominated by free-surface flow [23], carbonate dissolution is primarily governed by underground air transporting the necessary CO_2_ [24,25].

The movement of underground air strongly influences its role in storing and transporting chemical species and heat in karst. Consequently, underground air velocity—wind and ventilation—has been extensively studied [11,26,27], particularly regarding CO_2_ concentration [25,28,29], speleothem deposition, and paleoclimate records [30,31].

The wide-ranging effects of air movement are reflected in the various principles used to measure it [32,33]. Nevertheless, accurately measuring low air velocities remains challenging. The typical sensor of choice, the ultrasonic anemometer, is often fragile, power-intensive, or costly. While useful in cave settings, its deployment in challenging locations and over extended time periods may be impractical.

A potential alternative is a soft sensor. A soft sensor, also called a virtual or inferential sensor [34], is a computational model that estimates the value of an unmeasured variable. It uses measurements of interdependent variables and a process model to estimate the unmeasured variable, employing either first-principles or data-driven models. Soft sensors are typically used when variables are complex, expensive, or impossible to measure directly in real time. Soft sensors are widely applied in process engineering [35] and manufacturing [36]. Recent surveys cover this topic [37,38,39]. Although common in engineering, karst science has not yet adopted soft sensors.

In this study, wind sensors are replaced by a combination of other sensors and a mathematical model to estimate wind velocity and direction. Inputs to the soft sensor can include temperature measurements, which are easier to obtain in caves than wind, and surface meteorological variables measured or predicted near the cave. Because karst underground is typically poorly characterised, soft sensors generally rely on data-driven rather than first-principles models [40]. Developing a soft sensor for wind velocity, therefore, requires wind measurements over a calibration period. The key benefit of using a soft sensor is simpler maintenance of the measurement system. Alternatively, the soft sensor can operate alongside the physical anemometer, filling in the data gaps. Importantly, a soft sensor can be developed retrospectively for past periods with available input data. Temperatures in a cave passage can be monitored first to determine whether wind measurement would be beneficial. Once an anemometer is added, a soft sensor can be calibrated and used to reconstruct wind data for the earlier period of temperature monitoring.

The paper’s main original contributions are as follows:

- A data-driven method for developing a soft sensor of a meteorological variable in karst underground;
- Demonstration of the method’s effectiveness via a case study of wind in a selected cave passage.

The rest of the paper is structured as follows: Section 2 outlines the theoretical tools, Section 3 presents the case study and instrumentation, Section 4 shows results, Section 5 discusses them, and Section 6 provides a concise summary and concludes.

## 2. Methods

### 2.1. Procedure for the Development of a Soft Sensor

Developing a soft sensor follows a procedure similar to mathematical modelling. The main steps for the development are as follows:

- Selection of variables to model and defining the soft sensor requirements, such as accuracy and precision.
- Data collection and preprocessing.
- Modelling the system using methods such as experimental modelling (system identification), first-principles modelling, or observer design. The topic is well-documented in literature, e.g., [41,42].
- Validation of the soft sensor against preset requirements using data not used in the development of the soft sensor.
- Implementation on-site and regular maintenance.

Soft-sensor performance is highly dependent on measurements of other interdependent variables. Therefore, measurements must be consistent with field conditions, and equivalent sensor measurements should be used during development of the soft sensor and its operation. Any inconsistency leads to deviations in soft-sensor outputs. Examples of such discrepancies include sensor relocation or changes in the operational environment of the sensor.

Here, we focus on data-driven soft-sensor development using experimental modelling, also known as system identification. Any suitable data-driven modelling method, such as linear or nonlinear regression or classification, may be employed. We use a Gaussian process (GP) regression model to both obtain the prediction and quantify its uncertainty. Alternative models [43] may be various neural network models, such as multilayer perceptron models or long-short-term memory models, kernel models, such as support vector machines, fuzzy models, etc. Main strengths of the GP model in comparison with other models are flexibility because it can model complex nonlinear functions without specifying explicit structure; uncertainty quantification providing prediction variance; it performs well with small-to-moderate datasets; and others. The main weaknesses of the method are scalability and computational cost, where computational load rises with the third power of the number of data points, which can be circumvented using approximation methods.

### 2.2. Assumptions and Constraints

Observations used to develop the model underlying our soft sensor came from cave and surface measurements, supplemented with numerical weather forecasts. The latter are essential when weather measurements are not available. Sensor placement is fixed and must remain unchanged. Accurate instruments are essential because the soft sensor’s accuracy depends on them.

All variables are measured synchronously.

### 2.3. Performance Metrics

Modelling performance was evaluated using two cost functions. The first is selected to evaluate the model’s time-dependent predictions relative to the original system’s response. This evaluation uses the normalised mean-squared error (NMSE), defined as [44]

(1) $$\mathrm{NMSE}=\frac{1}{N}\frac{{∥y-E(\hat{y})∥}^{2}}{\sigma_{y}^{2}},$$

where

y—the vector of observations,
$E(\hat{y})$—the mean value of estimations $\hat{y}$, where $\hat{y}$ is considered as a stochastic variable, which gives different values at each repeated observation,
$\sigma_{y}^{2}$—the variance of observations,
*N*—the number of observations.

NMSE is a commonly used standardised measure for the accuracy of predicted mean values; an NMSE of 0 indicates a perfect model. The coefficient of determination $R^{2}$ can be derived from NMSE and is defined as [45]

(2) $$R^{2}=1-\frac{{∥y-E(\hat{y})∥}^{2}}{N\sigma_{y}^{2}}\cdot 100\%=\left(1-\mathrm{NMSE}\right)\cdot 100\%.$$

It ranges from 0% to 100%, with higher values indicating better performance.

The second measure is the Mean Standardised Log Loss (MSLL) [46],

(3) $$\begin{array}{ccc} \mathrm{MSLL} & = & \frac{1}{2N}\sum_{i=1}^{N}\left[\ln\left(\sigma_{i}^{2}\right)+\frac{{\left(E\left({\hat{y}}_{i}\right)-y_{i}\right)}^{2}}{\sigma_{i}^{2}}\right]\\ & - & \frac{1}{2N}\sum_{i=1}^{N}\left[\ln\left(\sigma_{y}^{2}\right)+\frac{{\left(y_{i}-E\left(y\right)\right)}^{2}}{\sigma_{y}^{2}}\right], \end{array}$$

where $\sigma_{i}^{2}$ is the variance of prediction in the *i*-th step, $\sigma_{y}^{2}$ is the variance of observations, y is the vector of the observations and E(·) denotes the expectation, i.e., the mean value.

MSLL is a standardised measure suitable for evaluating predictions of random variables. It incorporates the variance of predictions; prediction errors are weighted more heavily when associated prediction variance is smaller. The MSLL is approximately zero for the simpler models and negative for the more complex ones.

### 2.4. Gaussian Process Models

Gaussian process (GP) modelling [46,47], or kriging, models the input-output relationship f(x) of the regression vector x with a GP. A GP model is a nonparametric, probabilistic model used for regression, classification, and function approximation. It is particularly powerful when both the prediction and the associated uncertainty estimate are required. GP is a stochastic process containing random variables $f(x_{i})$ with a normal probability distribution,

(4) $$p\left(f\left(x_{1}\right),\ldots ,f\left(x_{N}\right)|x_{1},\ldots ,x_{N}\right))=N\left(m,K\right).$$

**Figure 1:**
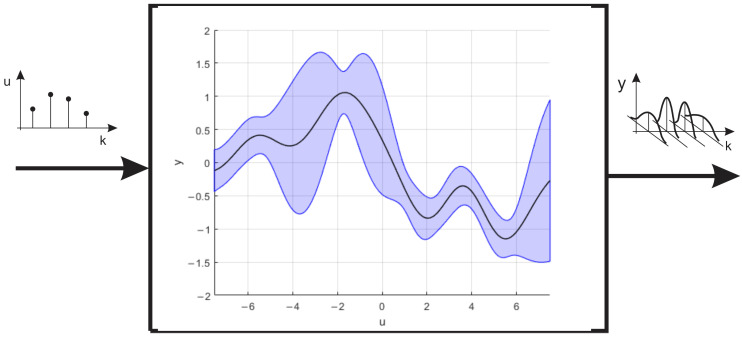
The principle of the Gaussian process model.

The vectors $x_{i}$ are regressor vectors, *f* denotes the GP, m is the mean vector and K is the covariance matrix of the Gaussian distribution N. In GP modelling, we describe the GP with a mean function and a covariance function,

(5) $$m_{i}=m\left(x_{i}\right),\;\;\;K_{ij}=C\left(x_{i},x_{j}\right),$$

where $m(x_{i})$ is the mean function and $C(x_{i},x_{j})$ is the covariance function. GP models flexibly approximate complex functions (Figure 1) using covariance kernels [47].

### 2.5. Models of Dynamic Systems

The system under study is dynamic. Its output depends on current and past inputs, unlike in a static system, where it depends only on current input. This consideration requires careful selection of the regression model structure. Various dynamic model structures can be employed for soft sensors. We focus on two commonly used models: the Finite Impulse Response (FIR) and the AutoRegressive model with exogenous input (ARX).

#### 2.5.1. Finite Impulse Response (FIR) and Autoregressive (ARX) Models

**Figure 2:**
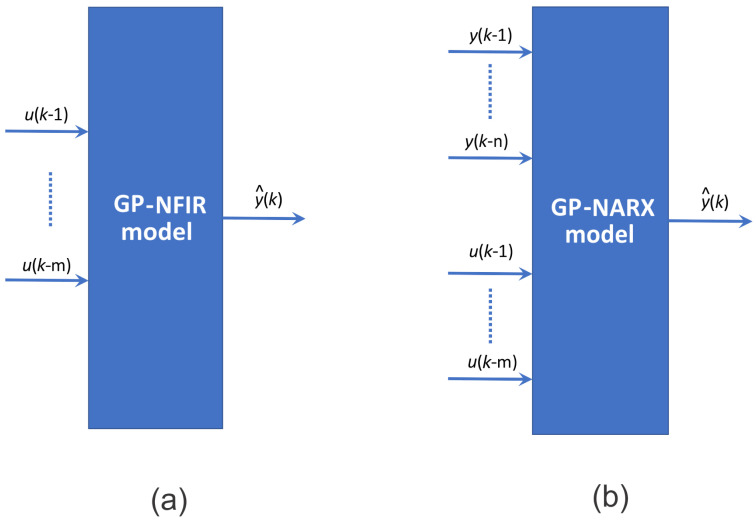
Two GP model structures: (**a**) GP-NFIR model, where the output predictions $\hat{y}$ are functions of *m* previous measurements of input signals *u*, (**b**) GP series-parallel or equation-error or NARX model, where the output predictions are functions of previous measurements of the input and output signals.

Nonlinear finite-impulse-response (NFIR) models employ solely the present and past samples of the input signal u∈R—specifically u(k) and u(k−i) at time step k∈N for i∈N—as regressors. Since regressors are restricted to input measurements only, NFIR structures are inherently stable, a crucial advantage when dealing with nonlinear systems where ensuring stability is more involved. The main advantages of NFIR models are already mentioned: stability, simplicity, and, consequently, fast training. A primary disadvantage is that they must frequently use many previous values of input variables to mimic the model dynamics, thereby increasing the model’s complexity. NFIR structures are well-suited to tasks such as control, identification of dynamical systems, noise suppression, modelling nonstationary time series, adaptive equalisation of communication channels, and various other signal-processing applications. A block diagram of the GP-NFIR model is shown in Figure 2.

Nonlinear ARX (NARX) models use both input values u(k−i);I,k∈N and measured output values y(k−i) as regressors. NARX formulation—also referred to as the equation-error or series–parallel representation—estimates the next output sample $\hat{y}\left(k\right)$ from the latest available lagged input and output measurements.

(6) $$\begin{array}{ccc} \hat{y}\left(k\right) & = & f(y(k-1),y(k-2),\ldots ,y(k-n),u(k-1),\\ & & u(k-2),\ldots ,u(k-m))+\nu , \end{array}$$

where *n* is the maximum lag in the output values, *m* is the maximum lag in the input values, and ν is the white Gaussian noise. The main advantages of NARX models are they are more compact, as they require fewer lags, and they can capture internal states of systems, yielding better predictive performance when systems have internal states. The main disadvantages are potential simulation instability due to feedback; estimation and simulation are much more challenging and often iterative because past outputs are used in the model; consequently, they are computationally more demanding; and they can be biassed when outputs are noisy. The GP-NARX model is schematically shown in Figure 2.

#### 2.5.2. What Are Prediction, Forecasting or Multi-Step Ahead Prediction, and Simulation?

One-step-ahead prediction (in short, prediction) denotes estimating the output at the immediate next sample. This is the standard task for prediction-type models such as ARX. The model’s long-term behaviour—used for forecasting over extended horizons or for validation—is typically assessed by simulation. Here, simulation means multistep-ahead prediction, where the horizon is either unbounded or matches the time span of interest for the analysis. For autoregressive schemes, multistep prediction can be performed by [47]:

- direct approaches, which train a distinct model for each target horizon, or
- iterative approaches, which obtain longer-horizon forecasts by repeatedly applying a one-step predictor.

The direct approach is limited because the prediction horizon must be chosen in advance and fixed; changing the horizon forces retraining. It also demands much more training data for strongly nonlinear systems that require long horizons. In the iterative formulation for Gaussian process predictors, the current output forecast is a function of prior output forecasts and observed inputs, expressed as:

(7) $$\hat{y}\left(k\right)=f\left(\hat{y}\left(k-1\right),\ldots ,\hat{y}\left(k-n\right),u\left(k-1\right),\ldots ,u\left(k-m\right)\right)+\nu \left(k\right),$$

where $\hat{y}\left(k-i\right);i=1\ldots n$ denotes the output estimate *i* samples or time steps in the past.

## 3. Case Study—Brezimeni Rov Passage in Postojna Cave, Slovenia

Measuring airflows in caves is crucial for understanding underground air dynamics. However, this is challenging because it requires sensitive, accurate anemometers, which are expensive and power-intensive. Using soft sensors can be a convenient and cost-effective alternative. There are also a couple of other reasons for using the soft sensor that are even more important in our study. The reasons are as follows:

- Due to the impracticality of anemometers, wind data are missing for some past periods when temperatures were recorded. We believe our site is no exception in this regard. Monitoring often begins with only the more convenient sensors, with advanced ones added if the site warrants further study. The only way to obtain the data on quantities that were not measured during the initial period is with soft sensors.
- Without real-time data connections, equipment failures are detected only during periodic visits, creating substantial data gaps, possibly months long. Most underground monitoring sites encounter this challenge. Depending on the type of study, such gaps may be a significant obstacle, but soft sensors can be used to fill them.

**Figure 3:**
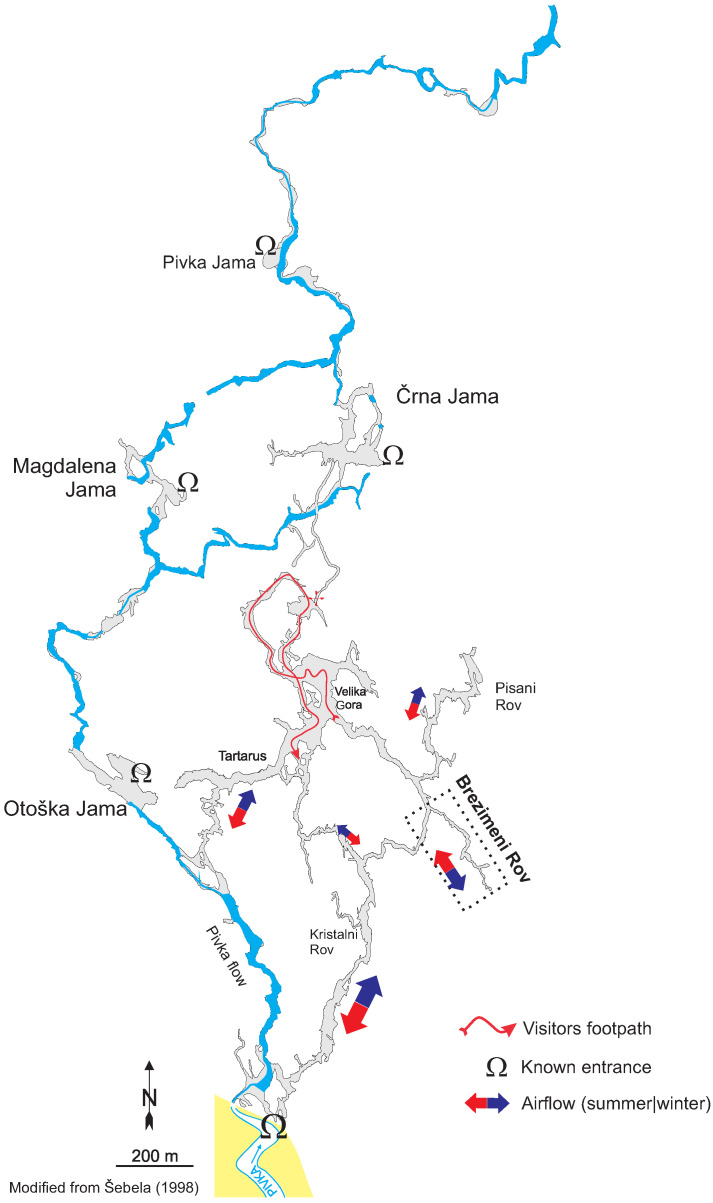
Position of the Brezimeni Rov passage within the Postojna Cave. Adapted from Šebela [53].

Postojna Cave, in Slovenia’s karst region, is a popular tourist attraction [48]. Its entrance is located at 45.783° N, 14.204° E, and its map is presented in Figure 3. The influence of the large number of visitors on the underground environment presents both a need for monitoring for conservation purposes and a research opportunity. Brezimeni Rov, marked in Figure 3, contributes to the ventilation of the most visited parts of the cave to a substantial amount and has electrical power available at its entrance. Thus, it is a particularly suitable location for monitoring the underground air.

Brezimeni Rov branches from the Stara Jama passage. It is approximately 325 m long along its axis and is relatively uniform with few splits and loops. The initial several tens of metres consist of a simple channel formed in bedrock, partly filled with sediments and flowstone. Occasional chambers with some collapse follow, until after 180 m, the passage rises into a collapsed hall approximately 20 m across. From the hall to the point 230 m from the entrance, one can progress along different levels. 222 m from the start of the passage, there is a dome, and at 250 m, the passage splits. The eastern part is 30 m long, narrow (1 × 2 m), and mostly horizontal, while the southern part is 75 m long and more diverse, with ascents and descents and lots of flowstone and sediment.

**Figure 4:**
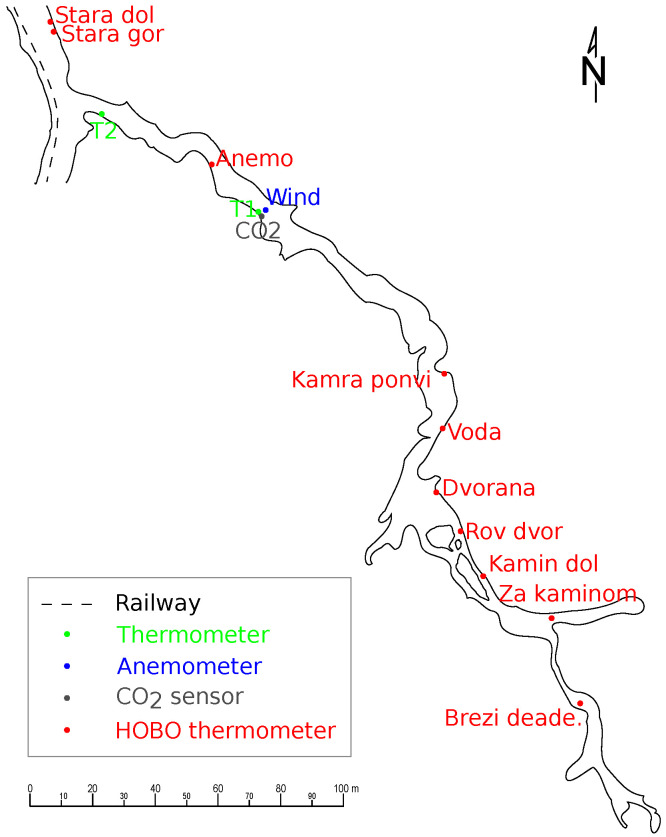
Brezimeni Rov passage with sensor locations. The dashed line in Stara Jama is the tourist railway. Drawn after Gallino [54].

Preliminary observations indicate that primary airflow through Brezimeni Rov occurs between the passage start and the dome at 222 m, from which we can infer that the dome has a connection to the surface that is passable for air. No additional air inflows or outflows were detected. The air flow through Brezimeni Rov is an important fraction of the total ventilation of Postojna Cave. Its intensity and direction imply that it is mainly driven by chimney effect: it has the direction into the passage and up the dome in cold periods, is reversed in hot periods, and is strongest at most extreme outside temperatures. Based on these observations, we decided to measure air velocity at a spot convenient for estimating air flow. Temperatures are measured at multiple points along the airflow path to quantify heat exchange between the rock and the air. Locations of sensors are shown in Figure 4.

**Figure 5:**
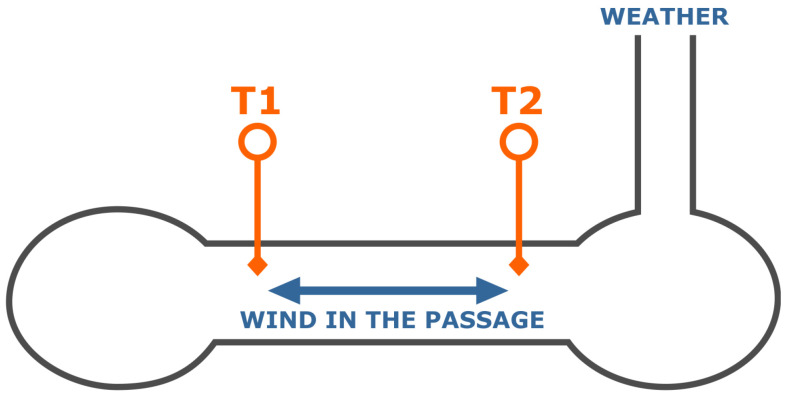
Scheme of conditions of the Brezimeni Rov passage. The arrows show wind direction.

The wind sensor used is an Ultrasonic Anemometer 3D manufactured by Thies Clima, sampling at 20 Hz. The measurements are averaged over 30 min periods. Because of the cave layout (Figure 5), wind direction is primarily constrained to the two directions along the passage. To simplify sensor development, wind velocity and direction were combined into a single signal, with positive and negative signs indicating direction.

CO_2_ concentration is measured with the Carbon Dioxide Probe GM252 by Vaisala Oyj, Helsinki, Finland, offering the range of 0–10,000 ppm CO_2_ with the accuracy of ±40 ppm CO_2_ at concentrations up to 3000 ppm and ±2% above. Temperature T1 is measured with a Pt-100 sensor pt100-1 by Microstep-MIS, Bratislava, Slovakia and T2 with a Pt-100 sensor HYGROCLIP S3C03-PT15 by Rotronic, Ettlingen, Germany both accurate to ±0.1 K. The temperatures are read and logged by the AMS-111 data logger from Microstep-MIS, Bratislava, Slovakia. Ten additional temperatures are recorded with autonomous loggers of the type MX2203 TidbiT MX Temperature 400’ Data Logger by HOBO, Onset Computer Corporation, Bourne, MA, USA, accurate to ±0.2 K and with resolution 0.01 K. The locations of the sensors are presented in Table 1.

The sensor ‘Temperature HOBO Brezi deade’ 277 m into the passage serves as a control, verifying that conditions beyond the dome at 222 m are stable with minimal changes in the weather.

The sensors ‘Temperature HOBO Stara dol’ and ‘Temperature HOBO Stara gor’ are located in the Stara Jama passage that the Brezimeni Rov passage connects to. They are north of the entrance to the Brezimeni Rov passage, that is, further away from the cave entrance and deeper into the massif. Ceiling height at this location is 7.7 m. The sensor’s Temperature HOBO Stara dol’ occasionally experiences flooding.

At the wind sensor site, the passage is approximately 6.2 m wide and 2.7 m high. The sensor is located 2.2 m from the passage wall, and its centre is 0.65 m above the passage floor.

Outside weather data were obtained from the Slovenian Environmental Agency (ARSO) station at 45.772° N, 14.197° E. The measurements of the Pivka River are taken at the sink at the Postojna Cave entrance, also by ARSO.

Assuming cave wind events relate to atmospheric conditions above Postojna Cave, regressors are chosen to maximise information about external conditions. The scheme of conditions of the Brezimeni Rov passage is illustrated in Figure 5.

The representativeness of the measurements at the Postojna ground-level meteorological station of the national measurement network is limited. In particular, wind measurements, carried out as standard at 10 m, can reflect a very local situation. The reason is that the surroundings of Postojna are characterised by very complex terrain. Weather forecasting offers an alternative to station measurements. The weather forecasting model generally operates on slightly larger horizontal spatial cells and therefore does not capture all terrain details. Consequently, wind direction and speed in a spatial cell represent the broader area, rather than the specific micro-location of the meteorological station, as direct measurements do.

Postojna Cave has several larger, locally distributed openings and presumably also many small openings through which the cave air is connected to the outer atmosphere. Thus, data on wind, temperature, and pressure should represent a wider area than that covered by the meteorological station.

Weather reanalysis from the MEIS company, produced with the WRF model [49,50], is used. The model simulations, in a setup used for weather forecasting, have also been successfully validated in the past at several locations across Slovenia, where ground measurements and vertical profiles of wind and temperature are available [51,52].

Reanalysis is an improved weather forecast that reconstructs past or current weather conditions without projecting into the future. It utilises measured weather data for boundary and initial conditions to reconstruct variables across the model’s entire 3D domain. This approach generally yields more accurate results than forecasting future weather.

Characteristics of the used reconstruction of meteorological variables of the external atmosphere are as follows:

- The model used is WRF [49,50].
- Two nested domains.
- The inner domain is the area of Slovenia with minimal surroundings.
- The inner domain has a horizontal spatial resolution of 4 km × 4 km.
- The temporal resolution is half an hour.

We use the results for a 4 km × 4 km ground cell covering the area around Postojna, where the main cave entrance is located. The data are treated as if a ground station were located in the centre of the cell, reflecting smoothed terrain characteristics rather than highly local variations. Reanalysis provides (the rest of the variables are listed in Table A2) the following:

- Air temperature at 2 m.
- Relative air humidity at 2 m.
- Wind speed and direction at 10 m.
- Air pressure at the ground.
- Short-wave incoming radiation.
- Precipitation.
- Cloud cover.

## 4. Results

### 4.1. Data

Data used for statistical modelling comprises cave signal measurements, weather station data from the surrounding area, and weather forecasts. Model output data are extracted from the signal combining wind velocity and direction (Table A1 in the Appendix A), while input data or regressors are obtained from the remaining signals, listed in Table A2 in the Appendix A.

The data points are separated by 30 min, which is the sampling rate of the weather forecast. The measurements are taken every 10 or 5 min, so they are downsampled; only the samples synchronous with the weather forecast are kept. The only signal that requires more preprocessing is the cumulative precipitation, where three 10 min totals are summed into each 30 min total.

These data were, according to modelling practice, divided into three sets: a training dataset, a validation dataset, and a test dataset. In modelling from data, ‘training data’ is the dataset used to teach the model by adjusting its parameters, ‘validation data’ is used during training to select structure, tune hyperparameters, and prevent overfitting, and ‘test data’ is a separate dataset used after training to evaluate the model’s final performance on unseen data.

While outside weather measurements and forecasts are often gap-free, this cannot always be said for cave measurements. Consequently, data should be preprocessed to remove the gaps. There are two options for eliminating the gaps. The first one is to use a data imputation [55], e.g., to interpolate, linearly or with other suitable functions, among the existing data, but this means that you incorporated some prior knowledge about how data should look in the gaps. Moreover, this option is only sensible when data gaps are narrow. The second option is to build the matrix of all regression vectors, called the regressor or feature matrix, for model training, and then eliminate rows with missing data to obtain a matrix with all elements. While this is the procedure for the training dataset, the test dataset should contain only time series without gaps, even though you limit the test data range. In our case, we selected a data range with no gaps for training, validation and test.

**Figure 6:**
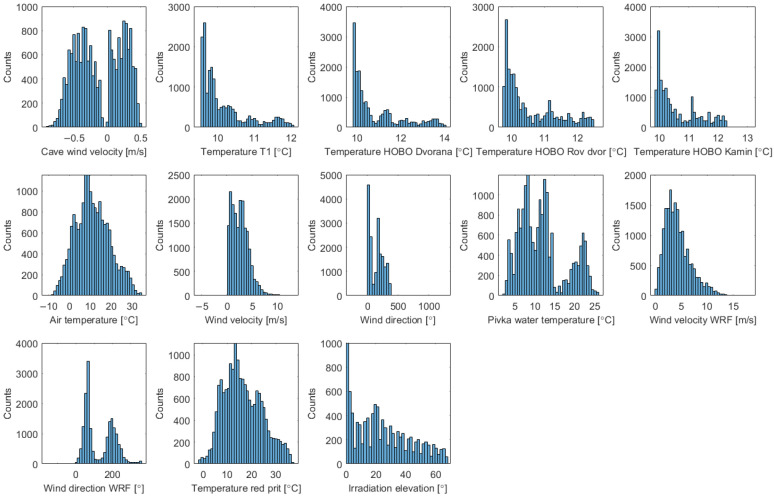
Histograms of data that were used for modelling, some for one and some for another model structure.

The dataset included approximately 18,000 points for training and validation, and 11,500 for testing. Examination of data distributions is beneficial for identifying anomalies, such as outliers or data points that, for various reasons, are outside the expected range. Figure 6 shows the distributions of signals used for modelling. The training signal (the reference wind velocity) consisted of data from 2024. The test signal comprised data from January 2025 until the end of August 2025. The test dataset is used solely for the model’s final evaluation.

The final step in data preprocessing is data normalisation, which yields scale alignment, improved numerical stability, faster and more stable optimisation, and other benefits. There are different ways to normalise data. We used standardisation, in which we scaled the input data to have a zero mean and unit variance. One needs to keep in mind that model responses must be denormalised before comparison with the original data.

### 4.2. Model Structure

Model structure selection depends partly on the chosen modelling method. Two model structures are compared: NFIR and NARX. For each modelling method, regressors have to be selected. The regressors, as well as other structural parameters, are determined using cross-validation. In our case, we used a 3-fold cross-validation, in which the data, excluding the test data, is divided into three equal subsets. Each subset is used once as a validation dataset, while the remaining two are used as a training dataset. The scores of statistical measures from three modelling runs are averaged, and the structure inputs or regressors with the best score are selected. The obtained results are presented below.

#### 4.2.1. Nonlinear Finite Impulse Response—NFIR Structure

The regressor-selection procedure, using the backward elimination method [47], yielded six signals, comprising measurements of variables taken both inside and outside the cave, as well as weather forecasts. These signals ranged from one to five lags to introduce dynamics into the model. Finally, 30 regressors were used as input to the NFIR model, as shown in Table A3 in the Appendix A, with one output representing cave wind velocity. The isotropic rational quadratic covariance function [47] was selected as the most appropriate based on cross-validation (Table A5 in Appendix A). Other parameters used for GP models (see [46,47] for details) include a zero mean function and exact inference and prediction with a Gaussian likelihood.

#### 4.2.2. Nonlinear AutoRegressive Model with Exogenous Input—NARX Structure

The regressor-selection procedure, employing the forward selection method [47], yielded nine signals, comprising measurements of variables taken inside and outside the cave, as well as weather forecasts. These signals ranged from one to two lags to introduce dynamics into the model. The number of lags and, consequently, the number of regressor candidates are smaller for the NARX model. Finally, 11 regressors were used as input to the NARX model, as shown in Table A4 in the Appendix A, with one output representing cave wind velocity. The isotropic Matérn covariance function with the smoothness parameter of value $\frac{3}{2}$ [47] was selected as most appropriate based on cross-validation (Table A5 in Appendix A). Other parameters used for GP models (see [46,47] for details) include a zero mean function and exact inference and prediction with a Gaussian likelihood.

### 4.3. Learning of the Model and Test Results

**Figure 7:**
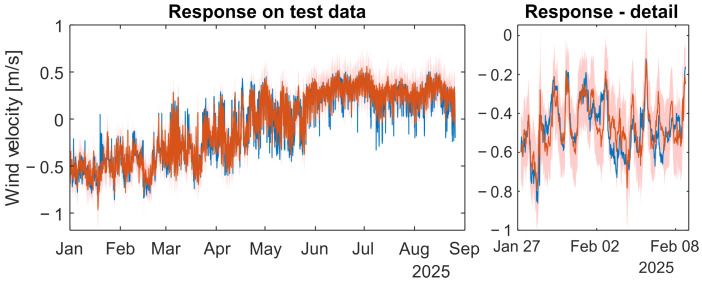
The simulation response of GP-NFIR wind-velocity model (train signals: complete year 2024)—red line with 95% confidence band compared with the measured test signal of the first eight months of year 2025—blue line.

**Figure 8:**
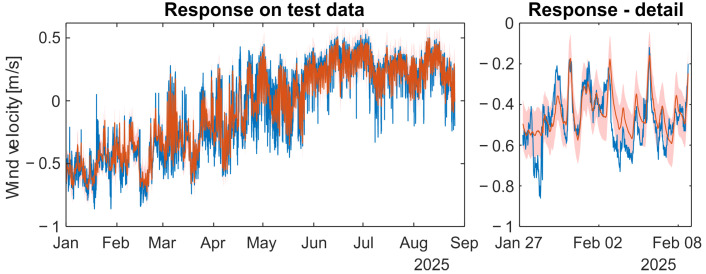
The simulation response of GP-NARX wind-velocity model (train signals: complete year 2024)—red line with 95% confidence band compared with the measured test signal of the first eight months of year 2025—blue line.

The full dataset, excluding test data, was used for model training. The MATLAB Statistics and Machine Learning Toolbox was used to train and test the models. The hyperparameters of models are given in Table A6 in the Appendix A. The obtained results are shown in Figure 7 for the NFIR model and in Figure 8 for the NARX model. In the NFIR model, there is no distinction between prediction and simulation because of its structure. The NARX model employs the iterative method to simulate the dynamic model. The statistical evaluation of simulation results on test signals yields NMSE = 0.056, R^2^ = 94.44% and MSLL = −1.437 for the GP-NFIR model, and NMSE = 0.066, R^2^ = 93.37% and MSLL = −1.113 for the GP-NARX model.

Suppose the simulation results are divided into absolute values of wind velocity and wind direction. In that case, the statistical measures for the simulated absolute values of wind velocity are NMSE = 0.216, R^2^ = 78.39% and MSLL = −0.738 for the GP-NFIR model, and NMSE = 0.246, R^2^ = 75.41% and MSLL = −0.568 for the GP-NARX model. The statistical evaluation of direction results yields NMSE = 0.092 and R^2^ = 90.83% for the GP-NFIR model, and NMSE = 0.128 and R^2^ = 87.24% for the GP-NARX model. For wind direction evaluation, data points with velocities below 0.1 m/s were excluded.

## 5. Discussion

Our goal was to develop a statistical model capable of substituting direct measurements of underground wind velocity. The model is considered effective if it can forecast the variable of interest using available measurements. The simulation results using independent data indicate that the goal has been achieved. No significant differences in simulation quality were observed between the model structures, as shown in Figure 7 and Figure 8. While statistical measures provide insight into model performance, visual inspection of all model responses remains essential. The evidence suggests that the resulting models are suitable for use as soft sensors.

Regressors obtained via a systematic machine-learning procedure differ between the two model structures. This difference confirms that the regressors provide statistical information to the model, rather than physics-based information. Physical causality should not be inferred from statistical models alone. But the hypothesis of physical causality can be confirmed using statistical methods. In this study, causality cannot be inferred from the obtained model, as indicated by the differing optimal regressors.

While the lists of selected regressors do not imply causality, they do match the general expectations. The regressors of the GP-NFIR model, listed in Table A3, are based on outside temperature, wind velocity, and wind direction, and on temperatures at three locations inside the Brezimeni Rov passage. Underground airflows are driven by the wind and by the temperature differences between the subsurface and the outside air [27], which are precisely the quantities captured by the selected regressors. The temperatures inside the cave are also affected by the cave airflow, which can further explain their relevance. Several lags are present due to the dynamics of the system—trends in these quantities affect the airflow as well. The signals underlying the regressors are of good quality, measured with sufficient accuracy, and distributions of their values cover the anticipated ranges, as demonstrated in Figure 6.

The regressors of the GP-NARX model are listed in Table A4. Past values of cave airflow are prominent; they encode information about the forces that previously drove the airflow and therefore serve as strong predictors of the current airflow. The other regressors that further improve the predictions contain the outside air temperature and wind, and a couple of air temperatures in the cave, just like in the case of GP-NFIR. In GP-NARX, the outside wind information is obtained from the reanalysis, while the source in GP-NFIR is the measurement. Although they originate from different sources, both pairs of signals contain similar information, making it difficult to explain the models’ differing preferences. Unlike in GP-NFIR, we also see regressors based on the forecasted potential temperature and sun elevation, and the measured temperature of the river that sinks into the cave. The potential temperature provides more information on the weather; the elevation of the Sun depends on the part of the day and the season. The river temperature both affects the cave airflow and provides information on the weather in the past. Additional studies would be necessary in order to determine whether the soft sensor benefits more from the river temperature’s direct thermal influence on cave airflow or from its role as a proxy for past weather conditions.

In our case, the simulation accuracy of both evaluated models, GP-FIR and GP-NARX, is quite similar. Nevertheless, one must consider the properties of each model, listed in Section 2.5, when selecting the model structure.

Statistical models are generally reliable for interpolation but not for extrapolation. Consequently, the model—and thus the soft sensor—is reliable only within the range of the training data. Beyond this range, model predictions may be inaccurate. In Bayesian models, including Gaussian process models, low confidence in model responses is indicated by high variance.

The close agreement between model responses and independent data supports the use of dynamic models. Although dynamic models are considerably more complex than static models, it is important to assess whether dynamics can be neglected.

By demonstrating the use of a statistical model to develop a soft sensor for underground wind velocity, we fulfilled the goal of showing how soft sensors can be utilised when hardware sensors are unavailable.

A common challenge in environmental monitoring is the occurrence of data gaps due to sensor failure [56]. Data on karst systems is no exception [57,58,59]. Such gaps may significantly bias statistics and impede long-term analyses [60]. In the case of Brezimeni Rov, heat balance studies cannot be conducted during periods with gaps in a quantity as crucial as wind velocity. The soft sensor enables such studies by providing estimates to fill data gaps.

Cave monitoring sites similar to our case study of Brezimeni Rov are widespread [18,61,62,63]. The choice of site for soft sensor development was not based on any special local characteristics. It can be inferred that applying this method at another well-instrumented site would produce a similarly successful soft sensor; hence, the approach can be recommended.

## 6. Conclusions

A data-driven soft sensor of wind velocity along a cave passage has been successfully implemented. It allows estimation of wind velocity in the passage from other measurements, without direct measurement. The constructed soft sensor demonstrates reasonable accuracy based on statistical measures and visual inspection of independent test data, supporting its use as a replacement for direct measurements.

When using a soft sensor, one must keep in mind that the data-driven model developed for the soft sensor cannot be applied to another location under any circumstances. The model remains invalid even after minor location changes. However, the method for developing a soft sensor demonstrated can be applied to any cave system at any location, regardless of key site characteristics, provided that key measurements are available. Some prior knowledge of cave-system physics is necessary to select appropriate variables for measurement, based on potential relationships with the soft-sensor output variable. A simple rule that more variables are better is applicable.

The demonstrated method has several advantages over direct measurement. A primary advantage is convenience, as most accurate wind sensors are expensive and power-intensive. Knowing wind velocity without measuring it is thus beneficial. Even with a physical sensor in place, a soft sensor can supplement missing data in the measurement time series. A soft sensor trained after installation of the physical sensor can also reconstruct past periods, provided the necessary input data were recorded.

Being data-driven, the soft sensor requires training data that includes the output quantity. The development of the soft sensor for wind measurement, therefore, requires that the physical anemometer be installed for a specific period. Only with sufficient training data can a soft sensor replacing the physical anemometer be developed.

This soft sensor will aid further research on cave meteorology and climatology. It can reconstruct wind speeds when no anemometer measurements exist and fill eventual data gaps. It may also allow downsizing of monitoring equipment; the anemometer could be removed while wind velocity continues to be obtained from the soft sensor. The present study serves as a proof-of-concept demonstrating the utility of soft sensors in karst science. More applications for various variables in underground systems are envisaged in future work, and these additional case studies will strengthen the proposed concept.

## Figures and Tables

**Table 1:** Sensors (symbol ‘x’ stands for check mark).

Name/Quantity	Location	Sampling Rate		Elevation
	**m From Start**	**30 min**	**5 min**	**m Above Ground**
Wind	73	x		0.65
CO_2_	73	x		0.3
Temperature T1	73	x		0.5
Temperature T2	12	x		0.6
Temperature HOBO Stara dol	−21		x	0.1 m above floor
Temperature HOBO Stara gor	−18		x	3.4 m above floor
Temperature HOBO Anemo	51		x	0.6
Temperature HOBO Kamra ponvi	150		x	0.5
Temperature HOBO Voda	166		x	underwater
Temperature HOBO Dvorana	192		x	0.5
Temperature HOBO Rov dvor	206		x	0.9
Temperature HOBO Kamin dol	222		x	1.4
Temperature HOBO Za kaminom	250		x	0.2
Temperature HOBO Brezi deade	277		x	1.1

## Data Availability

Dataset available on request from the authors.

## Appendix A. Available Signals, Selected Regressors and Covariance Function

**Table A1:** Signal/variable from which output data for training of the soft sensor is extracted (symbol ‘x’ stands for check mark).

Variable	Cave
Cave wind velocity (with incorporated direction)	x

**Table A2:** Signals/variables from which regressors were selected (symbol ‘x’ stands for check mark).

Variable	Cave	Outside	Forecast
CO_2_	x		
Temperature T1	x		
Temperature T2	x		
Temperature HOBO Stara dol	x		
Temperature HOBO Stara gor	x		
Temperature HOBO Anemo	x		
Temperature HOBO Kamra	x		
Temperature HOBO Voda	x		
Temperature HOBO Dvorana	x		
Temperature HOBO Rov dvor	x		
Temperature HOBO Kamin dol	x		
Temperature HOBO Za kaminom	x		
Temperature HOBO Brezi deade	x		
Air temperature		x	
Relative humidity		x	
Global radiation		x	
Diffuse sky radiation		x	
Pressure		x	
Wind velocity		x	
Wind direction		x	
Precipitation cumulative 10 min		x	
Snow height		x	
Pivka water temperature		x	
Pivka water level		x	
Precipitation cumulative		x	
Air temperature			x
Wind velocity WRF			x
Wind direction WRF			x
Precipitation cumulative			x
QNH			x
Cloudiness			x
Relative humidity			x
Global radiation			x
Diffuse sky radiation			x
Pressure			x
Temperature 1000			x
Temperature red prit			x
Irradiation direct			x
Irradiation global			x
Irradiation diffuse			x
Irradiation JDN			x
Irradiation elevation			x
Irradiation azimuth			x
Irradiation declination			x
Visibility TCDCclm			x
Visibility LCDClcll			x
Visibility MCDCmcll			x
Visibility HCDChcll			x
Visibility VISsfc			x

**Table A3:** Signals/variables and lags in samples selected as winning regressors in GP-NFIR model-structure optimisation.

Variable	Lag in Samples
Temperature T1	1
Temperature T1	2
Temperature T1	3
Temperature T1	4
Temperature T1	5
Temperature HOBO Rov dvor	1
Temperature HOBO Rov dvor	2
Temperature HOBO Rov dvor	3
Temperature HOBO Rov dvor	4
Temperature HOBO Rov dvor	5
Temperature HOBO Kamin dol	1
Temperature HOBO Kamin dol	2
Temperature HOBO Kamin dol	3
Temperature HOBO Kamin dol	4
Temperature HOBO Kamin dol	5
Air temperature	1
Air temperature	2
Air temperature	3
Air temperature	4
Air temperature	5
Wind velocity	1
Wind velocity	2
Wind velocity	3
Wind velocity	4
Wind velocity	5
Wind direction	1
Wind direction	2
Wind direction	3
Wind direction	4
Wind direction	5

**Table A4:** Signals/variables and lags in samples selected as winning regressors in GP-NARX model-structure optimisation.

Variable	Lag in Samples
Cave wind velocity	1
Cave wind velocity	2
Temperature HOBO Dvorana	2
Temperature HOBO Kamin dol	1
Air temperature	1
Air temperature	2
Pivka water temperature	1
Wind velocity WRF	2
Wind direction WRF	2
Temperature red prit	1
Irradiation elevation	2

**Table A5:** Mean-squared-error 3-fold cross-validation loss for covariance functions [47] during the selection procedure, where regressors as in Table A3 and Table A4 are used. ISO means isotropic covariance function, and ARD, which stands for Automatic Relevance Determination, means anisotropic covariance function. Loss values in bold are the winning values.

Covariance Function	GP-NFIR Loss	GP-NARX Loss
Matérn 3/2 ISO	0.037	**0.033**
Rational quadratic ISO	**0.033**	0.034
Rational quadratic ARD	0.040	0.035
Squared exponential ISO	0.048	0.034
Squared exponential ARD	0.046	0.034
Linear, one parameter ISO	0.194	0.035
Linear ISO	0.195	0.035
Linear ARD	0.195	0.035
Linear ARD + constant	0.195	0.035
Squared exponential ISO	0.051	0.034
Linear ISO + squared exponential ISO	0.038	0.035
Linear ISO + rational quadratic ISO	0.034	0.034
Lin. one parameter ISO + rational quadratic ISO	0.034	0.034

**Table A6:** Hyperparameters of GP-FIR and GP-NARX models.

Model	GP-FIR	GP-NARX
Covariance function	Rational quadratic ISO	Matérn 3/2 ISO
signal standard deviation	1.1	0.93
length scale	97	1.3
scale-mixture parameter	0.09	/
noise standard deviation	0.26	0.17
mean function	‘constant’	‘constant’
value	−0.66	−0.66
